# Antibacterial Activities of Ankaferd Hemostat (ABS) on Shiga Toxin-Producing Escherichia coli and Other Pathogens Significant in Foodborne Diseases

**DOI:** 10.4274/tjh.2015.0073

**Published:** 2017-03-01

**Authors:** Ahmet Koluman, Nejat Akar, İbrahim C. Haznedaroğlu

**Affiliations:** 1 Republic of Turkey Ministry of Food, National Food Reference Laboratory, Department of Mineral Analyses, Agriculture, and Livestock, Ankara, Turkey; 2 TOBB-ETU Hospital, Clinic of Pediatric Hematology, Ankara, Turkey; 3 Hacettepe University Faculty of Medicine, Department of Adult Hematology, Ankara, Turkey

**Keywords:** Ankaferd Blood Stopper, Shiga-toxigenic Escherichia coli, Salmonella, Campylobacter, Listeria monocytogenes

## Abstract

**Objective:**

Ankaferd hemostat (Ankaferd Blood Stopper^®^, ABS)-induced pharmacological modulation of essential erythroid proteins can cause vital erythroid aggregation via acting on fibrinogen gamma. Topical endoscopic ABS application is effective in the controlling of gastrointestinal (GI) system hemorrhages and/or infected GI wounds. Escherichia coli O157:H7, the predominant serotype of enterohemorrhagic E. coli, is a cause of both outbreaks and sporadic cases of hemorrhagic colitis. The aim of this study is to examine the effects of ABS on 6 different Shiga toxigenic *E. coli* serotypes including O26, O103, O104, O111, O145, and O157 and on other pathogens significant in foodborne diseases, such as *Salmonella* Typhimurium, *Campylobacter jejuni, and Listeria monocytogenes*, were also assessed.

**Materials and Methods:**

All strains were applied with different amounts of ABS and antimicrobial effect was screened. S. Typhimurium groups were screened for survival using the fluorescence in situ hybridization technique.

**Results:**

The relative efficacy of ABS solutions to achieve significant logarithmic reduction in foodborne pathogens *E. coli* O157:H7 and non-O157 serogroups and other emerging foodborne pathogens is demonstrated in this study. ABS has antibacterial effects.

**Conclusion:**

Our present study indicated for the first time that ABS may act against *E. coli* O157:H7, which is a cause of thrombotic thrombocytopenic purpura, hemolytic-uremic syndrome, and hemorrhagic colitis. The interrelationships between colitis, infection, and hemostasis within the context of ABS application should be further investigated in future studies.

## Abstract (tr)

**Amaç:**

Ankaferd hemostat (Ankaferd Blood Stopper^®^, ABS) gamma fibrinojene etki ederek eritroid agregasyonuna neden olan farmakolojik modülasyondur. Topikal endoskopik ABS uygulaması gastrointestinal (Gİ) kanamalarda ve enfekte Gİ yaralarında etkili olmaktadır. *Escherichia coli* O157:H7, en sık karşılaşılan enterohemorajik *Escherichia coli* tipi olup sporadik veya salgınlar şeklinde hemorajik kolitin önemli bir etkenidir. Bu çalışmanın amacı ABS ile 6 farklı Shiga Toksijenik *Escherichia coli* serotipi (O26, O103, O104, O111, O145 ve O157) ve diğer önemli gıda kaynaklı patojenlerden *Salmonella, Campylobacter* ve *Listeria monocytogenes* üzerine etkisi değerlendirilmiştir.

**Gereç ve Yöntemler:**

Tüm patojenler hazırlanarak ABS’nin farklı miktarları uygulanmış ve antimikrobiyel etki izlenmiştir. Salmonella canlılığı floresan in situ hibridizasyon tekniği ile izlenmiştir.

**Bulgular:**

ABS uygulamalarının sadece *Escherichia coli* O157 ve non-O157’ler üzerine değil aynı zamanda diğer patojenlerde de logaritmik azalma tetiklediği izlenmiştir. Bu çalışmada ABS ile farklı patojenler üzerine antibakteriyel etki gözlemlenmiştir.

**Sonuç:**

Bu çalışma özellikle trombositopenik purpura, hemolitik üremik sendrom ve hemorajik kolit yönünden önemli Escherichia coli O157:H7’nin üzerine ABS’nin antimikrobiyel etkisi olduğunu belirleyen ilk çalışmadır. ABS uygulamalarının kolitis, enfeksiyon ve hemostaz ilişkisi daha ileri seviyede araştırılmalıdır.

## INTRODUCTION

Ankaferd hemostat [Ankaferd Blood Stopper^®^, (ABS)]; http://www.ncbi.nlm.nih.gov/pubmed/?term=ankaferd) is the first topical hemostatic agent regarding red blood cell (RBC)-fibrinogen interactions tested in clinical trials [1]. ABS is composed of standardized plant extracts including *Alpinia officinarum, Glycyrrhiza glabra, Thymus vulgaris, Urtica dioica*, and *Vitis vinifera* [2]. ABS-induced pharmacological modulation of essential erythroid proteins (ankyrin, spectrin, actin) can cause vital erythroid aggregation by acting on fibrinogen gamma [3]. ABS also has pleiotropic effects, particularly in tissue healing, and has significant antiinfective properties [4,5,6,7,8]. The use of ABS in gastrointestinal (GI) system hemorrhages to control bleeding and/or infected GI wounds is also evident [9].

*Escherichia coli* O157:H7, the predominant serotype of enterohemorrhagic *E. coli* (EHEC), is a cause of both outbreaks and sporadic cases of hemorrhagic colitis [10]. Infection with E. coli O157:H7 presents with many complicated clinically abnormal hemostatic manifestations such as bloody diarrhea, hemolytic-uremic syndrome, or thrombotic thrombocytopenic purpura [11].

The aim of this study is to determine the effects of ABS on 6 different Shiga toxigenic *E. coli* (STEC) serotypes including O26, O103, O104, O111, O145, and O157. Moreover, the effects of ABS on other pathogens significant in foodborne diseases, such as *Salmonella* Typhimurium, *Campylobacter jejuni*, and *Listeria monocytogenes*, were also assessed. Elucidation of the effects of ABS on enterohemorrhagic bacteria is clinically important since there is a close pathobiological interrelationship between hemorrhages and hemostasis in terms of both diagnosis and management.

## MATERIALS AND METHODS

Thirty milliliters of ABS (Immune Drug Company, İstanbul, Turkey) was transferred to the laboratory under cold chain in a residue-free sterile tube. The sample was used for analyses within 30 min of arrival. Six different STEC serotypes, including O26, O103, O104, O111, O145, and O157 ATCC 43895 (obtained from Istituto Superiore di Sanita, Rome, and the Public Health Institution of Turkey), and *Salmonella typhimurium* ATCC 14028 (Microbiologics, UK), *Campylobacter jejuni* ATCC 33560 (Microbiologics, UK), and *Listeria monocytogenes* ATCC 19115 (Microbiologics, UK) were used in this study in order to assess the effects of ABS.

The cultures were stored at -80 °C. After thawing on ice, each strain (excluding *Campylobacter jejuni*) was incubated separately in 5x10 mL of brain-heart infusion (BHI) broth (Oxoid, UK) at 37 °C overnight. The cultures were passaged in BHI 3 times. The final cultures (5x10 mL) were centrifuged (Eppendorf) at 4200 rpm and 4 °C for 5 min. The supernatants were discarded, and pellets were resuspended and washed with 10 mL of sterile 0.9% NaCl. After washing, all suspensions were recentrifuged to remove organic residues. The resulting pellets were resuspended using sterile normal saline, and all strains were collected separately in a single tube. This stock culture was further diluted with 50 mL of sterile BHI broth to achieve a target level of 10^7^ to 10^8^ cfu/mL, which is accepted as sufficient for decontamination studies.

*Campylobacter jejuni* was streaked on 10 plates with charcoal cefoperazone deoxycholate modified agar (Oxoid, UK) with a sterile swab and incubated under microaerophilic conditions (Campygen, Oxoid, UK) at 42 °C for 48 h. The grayish colonies were collected into a centrifuge tube with a swab and the mixtures were centrifuged (Eppendorf) at 4200 rpm and 4 °C for 5 min. The supernatants were discarded, and pellets were resuspended and washed with 10 mL of sterile 0.9% NaCl. After washing, all pellets were recentrifuged to remove organic residues. The resulting pellets were resuspended using sterile normal saline, and all strains were collected separately in a single tube. This stock culture was further diluted with 50 mL of sterile Bolton broth (Oxoid, UK) to achieve a target level of 10^7^ to 10^8^ cfu/mL, which is accepted as sufficient for decontamination studies. All tubes were labeled and grouped into 2 separate groups. Tubes in group 1 were inoculated with 500 µL of ABS (per 50 mL, 1% v/v), and tubes in group 2 were inoculated with 1000 µL of ABS (per 50 mL, 2% v/v). All tubes were incubated at 37 °C under microaerophilic conditions to demonstrate the gut conditions, and samplings from these tubes were made at 5, 15, 30, and 60 min after inoculation. Next, 100 µL of these mixtures were spread-plated using a Spiral Plater (IUL, UK) on duplicate petri dishes of xylose lysine deoxycholate agar (Oxoid, UK) for *Salmonella*; MacConkey agar with sorbitol, cefixime, and tellurite agar (Oxoid, UK) for STEC; and chromogenic *Listeria* agar (Oxoid, UK) for *L. monocytogenes* and incubated at 37 °C aerobically for 24 h for all strains except *Campylobacter jejuni*, which was incubated under microaerophilic conditions (Campygen, Oxoid, UK) at 42 °C for 48 h. At the end of incubation period all typical colonies were counted and recorded.

*S*. Typhimurium groups were screened for survival using the fluorescence in situ hybridization (FISH) technique. Vermicon kits were used for this step. The study was composed of 3 independent trials and 9 tubes were analyzed at each step. The numbers of pathogens were converted to log_10_ cfu/g. The data were subjected to one-way analysis of variance (ANOVA) according to a (pathogen x treatment) 9x2 factorial design. The means were separated using Fisher’s least square differences method according to general linear models. Statistical significance level was accepted as 0.05. Statistical analyses were performed using Statistical Analysis System Software version 8 (SAS Inc., USA).

## RESULTS

The results indicating the effects of ABS on the studied bacteria are depicted in Tables 1 and 2. The relative efficacy of ABS solutions to achieve significant logarithmic reduction in foodborne pathogens *E. coli* O157:H7 and non-O157 serogroups and other emerging foodborne pathogens is also presented in Tables 1 and 2. According to the tables, 1% (v/v) application of ABS is not sufficient to obtain a significant decrease in the numbers of pathogens. On the contrary, 2% (v/v) application causes a dramatic decrease of the pathogens of concern. It was shown that by the end of the 60^th^ minute of application 2% (v/v) ABS causes a 4 log_10_ cfu/mL decrease, which was significant for all pathogens. The most significant decrease was recorded in *Campylobacter jejuni*, which is known for higher susceptibility to environmental and chemical changes.

**Figure 1:**
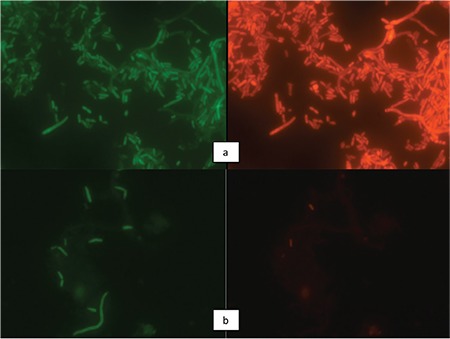
Effect of Ankaferd Blood Stopper, (ABS) on survival of S. Typhimurium (fluorescence in situ hybridization technique using Vermicon kit): a) Survival of S. Typhimurium with 2 mL of sterile distilled water at 37 °C. There is no visible change. Plating of the homogenate indicates the stability in the viable counts. b) Survival of S. Typhimurium with 2 mL of ABS at 37 °C. There is 3 log_10_ cfu/mL decrease, which indicates a statistical significance.

In Figure 1, photographs of two different applications on S. Typhimurium are provided. In the first group it can be clearly seen that sterile distilled water application had no effect on the survival of the pathogen. On the contrary, the second group of images clearly indicates the death of the pathogens with 2 mL of ABS.

## DISCUSSION

In this study, ABS was found to be effective against 6 different STEC serotypes, including O26, O103, O104, O111, O145, and O157, and *Salmonella typhimurium* and *Listeria monocytogenes*. Previous studies indicated that ABS might be used as a supportive agent together with antituberculous drugs during debridement of osteomyelitis and lymphadenitis lesions caused by multidrug-resistant *Mycobacterium tuberculosis* [4]. Oral/endoscopic ABS administration has already been performed in GI hemorrhages [9,12,13,14,15,16,17,18,19,20,21,22,23]. Moreover, ABS is active against multiresistant bacteria, such as methicillin-resistant *Staphylococcus aureus, Enterococcus* spp., generic *E. coli, Klebsiella* spp., *Acinetobacter* spp., and *Pseudomonas* spp., as well as fungi such as *Aspergillu*s spp., *Mucor* spp., and *Candida albicans* [4,5,6,7,8]. Our findings in this study further support previous research findings that ABS has antibacterial effects.

EHEC O157:H7 is associated with hemorrhagic colitis, thrombotic thrombocytopenic purpura, and hemolytic-uremic syndrome in humans [24]. EHEC O157:H7 infection can masquerade as GI bleeding of noninfectious cause, and the antecedent diarrhea may be resolved and forgotten by the time the hemolytic uremic syndrome or thrombotic thrombocytopenic purpura is diagnosed [25]. On the other hand, ABS represents an effective alternative treatment modality for GI bleeding, either as a primary or an adjuvant agent to conventional antihemorrhagic methods. The ABS GI data from published reports with encouraging results proved the safety and efficiency of ABS as a hemostatic agent for distinct states of GI bleeding. ABS is clinically effective in bleeding individuals with normal hemostatic parameters and in patients with deficient primary hemostasis and/or secondary hemostasis [9,12,13,14,15,16,17,18,19,20,21,23,26,27,28,29,30,31,32,33,34,35,36]. ABS may act as a topical biological response modifier as well as having antihemorrhagic actions. Our present study indicates for the first time that ABS may act against *E. coli* O157:H7, which is a cause of hemorrhagic colitis [10]. Difficult cases of infected bleeding radiation colitis have already been managed with ABS [9,19,36,37,38]. The interrelationships between colitis, infection, and hemostasis within the context of ABS application remain to be elucidated.

Infection, hemostasis, and wound healing are closely related pathobiological events to each other [39]. Next-generation RBC-related hemostatics, such as ABS nanohemostat, have been designated in the essential treatment of life-threatening bleedings by restoring physiological hemostasis via acting on RBCs [40]. Prohemostatic and antithrombin activities of ABS are linked to fibrinogen gamma chain and prothrombin by functional proteomic analyses. Those unique hemostatic properties of ABS provide a balanced hemostasis, representing a basis for physiological wound healing [3]. The proteomics of the structural and functional properties of the proteins related to the wound healing should also be matched with the already established proteomics of ABS [41]. Experimental trials indicated that ABS is effective in wound healing [39,42,43,44,45,46,47]. The results of our present study disclosed that ABS has antimicrobial effects against bacteria that are active in wound and burn complications.

The use of plant extracts and phytochemicals with established antimicrobial properties could be of great significance in preventive and/or therapeutic approaches. The increasing prevalence of multidrug-resistant strains of bacteria and the recent appearance of strains with reduced susceptibility to antibiotics raised the specter of “untreatable” bacterial infections and adds urgency to the search for new infection-fighting strategies. Besides broad-spectrum activity against gram-positive and gram-negative bacteria, including human pathogens and food-spoilage bacteria, ABS was found to be more stable than nisin in different heat and enzyme treatments by Akkoç et al. [5,48]. Furthermore, as indicated by Akkoç et al., the antibacterial activity of ABS can proceed in extreme environmental conditions such as the potential use of the preparation for the therapy of infectious diseases and preservation of different types of foods from foodborne pathogens or food-spoilage bacteria [5,48]. Our present results support the idea that the antiinfective properties of ABS should be tested in in vivo experiments [4,5,6,7,8].

The mechanism of action regarding the antiinfective actions of ABS is currently unknown. Several proteins (Homo sapiens malic enzyme 1, dynactin 5, cofilin, utrophin, mucin16 (CD164-sialomucin-like-2 protein), chalcone flavanone isomerase 1, chalcone flavanone isomerase 2, helezonal bundle transporter protein-141, hypothetical protein LOC283638 isoform 1, hypothetical protein LOC283638 isoform 2, complex 1 intermedia related protein 30) in ABS functional proteomic analyses represent an important step to elucidate how ABS biologically affects the components of numerous pathogens [41]. Comparative molecular studies covering proteomics, genomics, transcriptomics, and metabolomics of ABS are essentially important to shed light on this extremely vital area.

## CONCLUSION

The pleiotropic effects of ABS on the vascular endothelium, blood cells, angiogenesis, cellular proliferation, vascular dynamics, and cellular mediators should be investigated to determine its potential role in many pathological states, including infectious diseases, wound healing, and inflammation. ABS, as a unique hemostatic agent within many crossroads of hemostasis, infection, and neoplasia, casts future experimental and clinical research to be placed into clinical management.

## Figures and Tables

**Table 1:**
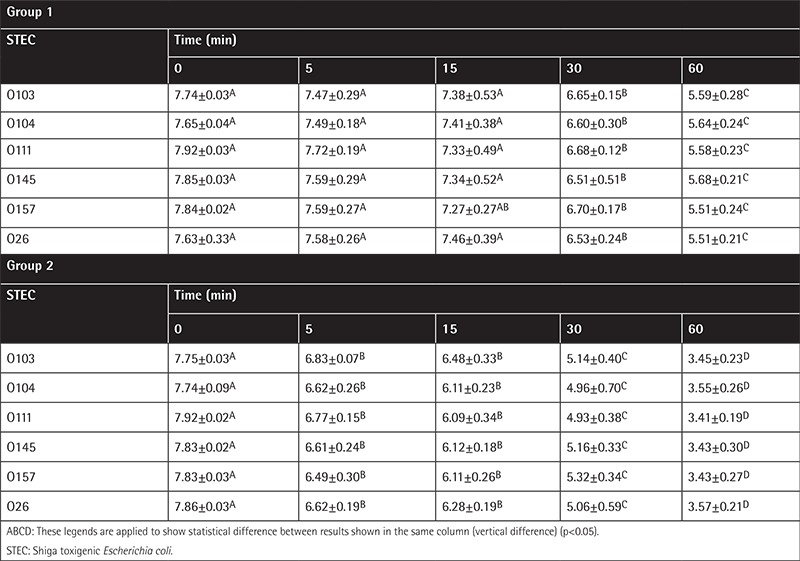
The Shiga toxigenic *Escherichia coli* results of the study in group 1 (sterile distilled water application) and group 2 [Ankaferd hemostat (ABS) application].

**Table 2:**
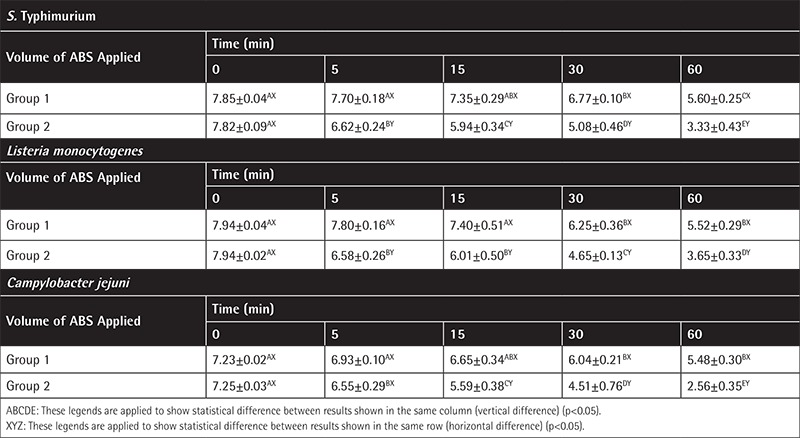
The in vitro results regarding *Salmonella* Typhimurium, *Listeria monocytogenes*, and *Campylobacter jejuni* in group 1 (sterile distilled water application) and group 2 [Ankaferd hemostat (ABS) application].
